# The effect of oxysterol imbalance in synaptic dysfunction

**DOI:** 10.1002/alz70855_105023

**Published:** 2025-12-24

**Authors:** Raúl Loera‐Valencia

**Affiliations:** ^1^ ITESM, Chihuahua, CI, Mexico

## Abstract

**Background:**

Oxysterols, particularly 27‐hydroxycholesterol (27‐OHC), have been implicated in neurodegenerative diseases by disrupting neuronal and astrocytic function. The synaptic proteins SNAP25 and PSD95 are essential for synaptic integrity, while REST/PTBP1 plays a key role in neuronal plasticity. In astrocytes, RAGE activation and the downregulation of glutamate transporters GLT‐1 and GLAST contribute to excitotoxicity. However, the precise molecular mechanisms underlying 27‐OHC‐induced neurotoxicity remain poorly understood.

**Method:**

Primary cortical neurons and astrocytes were exposed to physiological and pathological concentrations of 27‐OHC. Western blot, qPCR, and immunocytochemistry were used to assess the expression of SNAP25, PSD95, REST, PTBP1, RAGE, GLT‐1, and GLAST. Functional assays included calcium imaging and glutamate uptake measurements to evaluate synaptic and astrocytic responses.

**Result:**

Neuronal exposure to 27‐OHC led to a significant reduction in SNAP25 and PSD95 expression, correlating with synaptic dysfunction. Additionally, REST was upregulated, while PTBP1 was downregulated, suggesting impaired neuronal plasticity. In astrocytes, 27‐OHC induced RAGE upregulation and a marked decrease in GLT‐1 and GLAST levels, leading to reduced glutamate uptake and increased extracellular glutamate concentrations. These findings indicate a dual mechanism whereby 27‐OHC disrupts synaptic integrity in neurons while impairing astrocytic glutamate clearance, contributing to excitotoxicity.

**Conclusion:**

Our study highlights the detrimental effects of 27‐OHC on neuronal and astrocytic function, identifying key molecular targets involved in synaptic integrity and glutamate homeostasis. The observed dysregulation of SNAP25, PSD95, REST/PTBP1, and astrocytic RAGE, GLT‐1, and GLAST underscores the role of oxysterol‐induced neurotoxicity in neurodegenerative processes. Targeting these pathways may offer novel therapeutic strategies for conditions characterized by cholesterol dyshomeostasis and excitotoxicity.

